# The Voluntary Principle and the Bill

**Published:** 1911-08-05

**Authors:** Richard J. Coles

**Affiliations:** Secretary-Superintendent. Addenbrooke's Hospital, Cambridge.


### THE VOLUNTARY PRINCIPLE AND THE BILL.

*To the Editor of* The Hospital.

Sir,—I have received a copy of the report of a meeting of representatives of Charitable Institutions and Societies hereon, and I am extremely sorry to see that it is still being urged by those who profess to desire that the voluntary hospital system in the United Kingdom should be retained, that the Chancellor of the Exchequer should be pressed to make provisions in the Bill for hospitals to be paid for actual work done.

It seems almost incredible that those who not only desire the preservation of our voluntary hospitals, but express their willingness to fight for that cause, should be the first to urge that which can end in the greatest of calamities—i.e., the nationalisation of our hospitals.

It matters very little whether a contribution from the State takes the form of a grant on general lines or payment for work done, it is none the less "State aid," and with it must come "State control." These two are inseparable.

If provision is made in the Bill for the insured's sick allowance to be paid to the hospital during the time he is an in-patient at that institution, even that would be "State aid" under another name, and would not in any way reimburse the hospital for the cost of his maintenance.

Surely this sick allowance should go to the insured's dependants and not to the hospital.

For hospitals to accept such payments would be unwise.

Besides wrecking the most noble of institutions or foundations in our land, our voluntary hospitals, there would of necessity be the wrecking of that splendid incentive upon which our hospitals have not only been founded and maintained for the past two hundred years at least.

The voluntary spirit has been, and will continue to be, the very soul of our hospitals, which have always been, and are to-day, second to none in the whole world.

This "incentive" or "spirit" we know full well has played a most important part in moulding for good—in fact for all that is sterling—the character of the individual and the nation alike.

. . . . . . . . . .

Yours very faithfully, Richard J. Coles,
Secretary-Superintendent.

**Addenbrooke's Hospital, Cambridge.**

Mr. Coles has evidently not studied the Bill nor made himself familiar with the effect of the amendments to it which we have advocated. He cannot have realised either the grievous financial dangers in its original form which it must have inflicted upon many voluntary hospitals throughout the United Kingdom. We have dealt with the position as it in fact is to-day in the leading article this week. We shall welcome discussion on the subject from all hospital workers who may feel moved to express their views and experience.—Ed., The Hospital.

